# Development and Validation of Verbal Emotion Vignettes in Portuguese, English, and German

**DOI:** 10.3389/fpsyg.2019.01135

**Published:** 2019-06-14

**Authors:** Tanja S. H. Wingenbach, Leticia Y. Morello, Ana L. Hack, Paulo S. Boggio

**Affiliations:** Social and Cognitive Neuroscience Laboratory, Centre for Biological and Health Sciences, Mackenzie Presbyterian University, São Paulo, Brazil

**Keywords:** emotion vignettes, emotion, German, Portuguese, English

## Abstract

Everyday human social interaction involves sharing experiences verbally and these experiences often include emotional content. Providing this context generally leads to the experience of emotions in the conversation partner. However, most emotion elicitation stimulus sets are based on images or film-sequences providing visual and/or auditory emotion cues. To assimilate what occurs within social interactions, the current study aimed at creating and validating verbal emotion vignettes as stimulus set to elicit emotions (anger, disgust, fear, sadness, happiness, gratitude, guilt, and neutral). Participants had to mentally immerse themselves in 40 vignettes and state which emotion they experienced next to the intensity of this emotion. The vignettes were validated on a large sample of native Portuguese-speakers (*N* = 229), but also on native English-speaking (*N* = 59), and native German-speaking (*N* = 50) samples to maximise applicability of the vignettes. Hierarchical cluster analyses showed that the vignettes mapped clearly on their target emotion categories in all three languages. The final stimulus sets each include 4 vignettes per emotion category plus 1 additional vignette per emotion category which can be used for task familiarisation procedures within research. The high agreement rates on the experienced emotion in combination with the medium to large intensity ratings in all three languages suggest that the stimulus sets are suitable for application in emotion research (e.g., emotion recognition or emotion elicitation).

## Introduction

The everyday life of humans involves many social interactions which are rarely free of emotional content. When we interact with each other, we tell stories about experiences including emotional states, and use facial expressions to communicate about our emotional states in addition to varying intonation and speed of our speech. Thus, a multitude of stimulus sets providing sensory cues exist for investigation of related research questions, e.g., stimulus sets of facial emotion (literature review by Ekman and Friesen, 1976; Langner et al., 2010; Krumhuber et al., 2013; Wingenbach et al., 2016) and vocalisations (Belin et al., 2008) but also including multiple modalities (Bänziger et al., 2009, 2012; Hawk et al., 2009; Dyck, 2012). Such stimulus sets are useful when investigating participants’ processing of other’s emotions based on sensory information and are generally stripped of contextual information.

Stimuli including contextual information are more likely to elicit an emotion in the observer or listener. Stimulus sets have accordingly been developed with the purpose to elicit emotions. A widely used stimulus set is the International Affect Picture Set (IAPS; Lang et al., 1997) which includes thousands of images depicting emotional scenes validated to elicit affect ranging in valence from negative to positive (Ito et al., 1998). There are also dynamic stimulus sets that can elicit affect, e.g., a film-based stimulus set containing 20 stimuli of positive vs. negative social interactions (Carvalho et al., 2012). Whereas these stimulus sets range on the valence dimension, there are also stimulus sets that aim at the elicitation of specific emotions, e.g., emotion eliciting film sequences (McHugo et al., 1982; Philippot, 1993; Gross and Levenson, 1995; Schaefer et al., 2010).

Emotion-specific stimulus sets often include the six emotion categories which are agreed upon by most researchers to represent so called basic emotions (Ekman et al., 1969; Ekman and Cordaro, 2011), but see also (Ortony and Turner, 1990). These emotions are anger, disgust, sadness, fear, happiness, and surprise. Because these emotions are considered universal, i.e., culturally independent, their inclusion in stimulus sets is often standard. However, many more emotions exist and are often called complex emotions, since they include a greater cognitive component than basic emotions. Examples of complex emotions are gratitude and guilt. To be able to experience gratitude, it is necessary to evaluate an action by someone else as beneficial to oneself and costly to the other person at the same time (McCullough et al., 2008). It is this saccade of appraisals that makes gratitude a complex emotion. The same applies to guilt. Here, an action carried out by oneself might have been beneficial to oneself but included negative aspects for another person (Tracy and Robins, 2006). Guilt as well as gratitude are emotions that emerge in interpersonal contexts and are thus of great interest to social psychology research. The authors are unaware of a stimulus set suitable for elicitation of emotions including these two complex emotions next to basic emotions. It is possible that it is difficult to induce guilt and gratitude with images whether static or dynamic and that therefore the focus is on basic emotions within such stimulus sets.

As opposed to watching films or images, reporting about experiences in conversations within social interactions includes verbal descriptions of scenarios. A semantic understanding by the listener is required as well as abilities of perspective taking to understand the emotional experience of the narrator and to experience their emotions. Verbal vignettes depicting brief situations of emotional content are a useful research tool incorporating these aspects. The “Geneva Emotion Knowledge test – Blends” includes 28 verbal vignettes each portraying two out of 16 target emotions (pride, joy, happiness, pleasure, interest, anxiety, sadness, irritation, fear, disgust, anger, guilt, shame, contempt, jealousy, and surprise). These vignettes can be used to measure emotion understanding (Schlegel and Scherer, 2017). When participants are instructed to mentally immerse themselves in the described scenarios, it is possible to elicit emotion experience. For example, a published study taking this approach included one verbal vignette depicting five emotions (anger, sadness, jealousy, embarrassment, and anxiety) (Vine et al., 2018). Whereas the individual vignettes used by Schlegel and Scherer (2017) and Vine et al. (2018) included several target emotions, it is also possible to target specific emotions one at a time within individual vignettes.

Verbal vignettes describing situations of one target emotion each (anger, sadness, and fear) were created by MacCann and Roberts (2008) and Hareli et al. (2011), the latter included vignettes depicting guilt. The International Survey on Emotion Antecedents and Reactions (Scherer and Wallbott) is a database of situations described by almost 3000 participants that elicited a specific emotion in them (joy, fear, anger, sadness, disgust, shame, and guilt). Whereas guilt as a target emotion is sometimes included alongside other emotions, vignettes targeting gratitude are generally not included. However, there is published research which focussed on gratitude itself. For example, a study included three gratitude vignettes although two of these vignettes described the same situation but was varied in the intensity of the received benefit (Wood et al., 2008) and another study included 12 gratitude vignettes (Lane and Anderson, 1976). The authors are unaware of a vignette stimulus set including gratitude and guilt next to basic emotions.

The current research aimed at developing and validating verbal emotion vignettes of seven different emotion categories alongside neutral vignettes. To assure that the vignettes can induce emotions, high agreement rates on the experienced emotions, and intensity ratings were necessary. Thus, agreement rates and intensity rates were calculated per vignette. It was required for each individual vignette to distinctively map onto one emotion category based on the agreement rates, which was addressed with hierarchical clustering. Based on the agreement rates, hit rates (raw and unbiased), and intensity rates were calculated for each emotion category for comparison to published instruments. To increase the benefit of the emotion vignettes to the research community, the vignettes were created, and validated in three languages (Portuguese, English, and German).

## Materials and Methods

### Stimuli Creation

Verbal vignettes were created written from a first-person perspective to facilitate for the reader to imagine the situation described in the vignettes. The vignettes were each written with a similar length of ∼3 lines. It was aimed to describe scenarios that would clearly map onto one distinct emotion category. Initially, 10 vignettes were created per emotion category (anger, disgust, fear, sadness, guilt, happiness, and gratitude) and also for neutral scenarios. Several pilot studies were conducted on psychology student samples. Each pilot study led to adjustments of the wording of the vignettes and clarification of the task instructions with the aim to increase recognition rates of the individual vignettes. Every vignette with a recognition rate of the target emotion <80% was re-written to be more distinct. Eventually, 5 vignettes per emotion category with recognition rates of > = 80% were selected to be included in the validation study (presented in the results section of the current manuscript). The vignettes with the highest recognition rates were selected, as the aim for the vignettes was to have as little ambiguity as possible. All 40 vignettes in each of the three languages can be found in the Supplementary Material but example vignettes (one for each emotion category) are provided in the following:

Anger: “I was eating cake at home with my sister when her boyfriend arrived. He glanced at the cake and said she should stop eating because she was getting too fat and he wouldn’t date her anymore if she continued like that.”

Disgust: “On my way home, I saw a dead rat on the sidewalk. When I got closer I noticed its belly was open, decomposing, with tons of white maggots crawling inside it, and some coming out of its mouth.”

Fear: “It was late one night, and I was in a deserted plaza with some friends. We were laughing and walking in the direction of the car when my friend was struck in the back. We all froze when we saw two men pointing guns at us.”

Sadness: “When me and my sister were younger, we became orphans. We ended up being sent to different homes. I remember this day, because my sister cried a lot and held me tight. I didn’t understand why I couldn’t stay with her.”

Guilt: “When I ended my relationship, I shared intimate photos of my ex-girlfriend with a group of friends. These pictures were leaked to the internet, and afterward I found out she had been fired from her job for getting a bad reputation. I should never have done that.”

Neutral: “I left college at noon and went to the parking lot to pick up my car and leave. On the way, there was a restaurant and I had lunch there before heading on. I got on my way and home at two o’clock.”

Happiness: “I went to see a show of a band I’ve been a fan of since I was a teenager. During the show, the vocalist saw my poster, walked toward me smiling, and reached out to me while singing my favourite song.”

Gratitude: “Late one night, I slept on the last bus and only woke up at the final bus stop. My cell phone battery was dead and, hearing my story, a station worker let me borrow his phone to call someone.”

### Participants

Portuguese-speaking participants were recruited from the Mackenzie Presbyterian University student population through social media. Data was collected from 301 participants. A control measure was inserted in the online assessment to identify participants who did not pay attention to their answering. After exclusion of these individuals, the final sample size included in the analyses was *N* = 229 [202 females, 27 males; *M*(age) = 20.7 years, *SD* = 4.7]. English-speaking participants [*N* = 59, 30 females, 29 males; *M*(age) = 34.5 years, *SD* = 10.9] were recruited through social media from the general population. English as mother tongue was required for participation in the study. German-speaking participants [*N* = 50, 28 females, 22 males, *M*(age) = 37.4 years, *SD* = 11.7] were recruited from the general population through social media and German as mother tongue was a requirement for study participation. No participants were excluded from the English-speaking and German-speaking samples for analyses.

### Procedure

Ethical approval of the study was provided by the Mackenzie Presbyterian University Ethics Committee. Participants accessed the vignettes through a Google Forms survey and written informed consent for participation was obtained within the survey. Participants were instructed to participate from a place without distractions, to answer on their own, and not to engage in any other activity while completing the study. The instruction for each vignette was for the reader to imagine to be the person depicted in the scenario and immerse themselves in the scenario. Participants then had to choose one emotion category from a list of provided labels (one for each of the 8 emotion categories) to state what they were feeling while they imaged to experience the situation depicted in the vignette. Next, participants had to rate the intensity of the chosen emotion for the respective vignette on a 10-point Likert-scale ranging from 0 (=very low) to 9 (=very high). Completing the study took approximately 25 min. Portuguese-speaking participants were granted course credit for participation. English-speaking and German-speaking participants were not compensated for participation as required by Brazilian law.

### Statistical Methods

Data files (one for each language) were created including participants’ responses to each vignette. The responses to the first question (emotion label attributions) for each vignette were transformed to reflect target emotion attributions by assigning ones and non-target attributions by assigning zeros to be able to calculate raw hit rates per vignette (separately for each language). That is, for each vignette, the number of attributions of the target emotion across participants was summed, divided by the respective *N*, and multiplicated by 100 (i.e., rule of three, to represent percentages for ease of interpretation). Likewise, mean intensity rates (in %) per vignette were calculated (only considering classifications of the target emotion to the individual vignettes) by applying the rule of three, i.e., the intensity ratings of all participants were averaged per vignette, divided by 9, and multiplicated by 100.

Statistical analyses were conducted using the software SPSS (version 24; IBM Corp, 2016). A hierarchical cluster analysis with average linkage between groups and squared Euclidian distance was conducted (separately for each language) including all 40 vignettes to test whether the individual vignettes clearly mapped onto one emotion category as intended based on the sum of emotion label attributions per category (anger, disgust, fear, sadness, guilt, neutral, happiness, and gratitude). Vignettes that did not clearly map onto their target emotion category were eliminated and the hierarchical cluster analysis was conducted again only including the remaining vignettes.

Afterward, raw hit rates per emotion category were calculated (separately for each language) by averaging the raw hit rates (in %) of the four vignettes per emotion category to be included in the final stimuli sets as identified by the cluster analyses.

As a measure of distinctiveness, unbiased hit rates (Hu; Wagner, 1993) were calculated for each emotion category (separately for each language). *Hu* takes response biases into consideration by which the raw hit rates are corrected. The formula is *Hu* = a^2^/(a + b + c)*(a + d + e) where *a* represents the target emotion, *b* and *c* represent the misattributions of another emotion to the presented target emotion, and *d* and *e* represent the misattributions of the target emotion to other emotion categories. The resulting *Hu* rates represent percentages.

Intensity rates were calculated per emotion category (separately for each language) by averaging the intensity rates (in %) of the four vignettes per emotion category as identified by the cluster analyses to be included in the final stimuli sets.

## Results

### Portuguese Vignettes

Table 1 displays the *M*s and *SD*s of the raw hit rates and intensity rates for the individual vignettes.

**TABLE 1:** Agreement rates and intensity rates in percentages for each vignette in English, Portuguese, and German.

		Anger				Disgust				Fear				Sadness		
	#	ENG *M* (*SD*)	POR *M* (*SD*)	GER *M* (*SD*)	#	ENG *M* (*SD*)	POR *M* (*SD*)	GER *M* (*SD*)	#	ENG *M* (*SD*)	POR *M* (*SD*)	GER *M* (*SD*)	#	ENG *M* (*SD*)	POR *M* (*SD*)	GER *M* (*SD*)
**A** **I**	**1**	71 (46) 71 (18)	95 (21) 80 (22)	92 (27) 68 (22)	**6**	83 (38) 79 (23)	87 (34) 82 (20)	84 (37) 72 (22)	**11**	90 (31) 87 (20)	97 (16) 94 (13)	96 (20) 84 (23)	**16**	81 (39) 81 (24)	89 (31) 88 (20)	78 (42) 83 (25)
**A** **I**	**2**	71 (46) 81 (20)	91 (29) 87 (18)	88 (38) 75 (24)	**7**	80 (41) 76 (22)	87 (34) 73 (22)	78 (42) 65 (24)	**12**	86 (35) 86 (24)	97 (17) 92 (16)	84 (37) 83 (22)	**17**	80 (41) 82 (21)	88 (33) 85 (21)	78 (42) 79 (25)
**A** **I**	**3**	68 (47) 74 (21)	86 (35) 90 (15)	92 (27) 71 (20)	**8**	81 (39) 57 (25)	92 (28) 79 (24)	64 (49) 58 (24)	**13**	86 (35) 66 (25)	94 (23) 80 (21)	88 (33) 70 (22)	**18**	76 (43) 85 (20)	86 (34) 91 (17)	70 (46) 81 (22)
**A** **I**	**4**	64 (48) 59 (25)	72 (45) 73 (24)	80 (40) 65 (20)	**9**	81 (39) 81 (22)	94 (23) 87 (20)	88 (33) 79 (19)	**14**	81 (39) 84 (23)	91 (28) 95 (11)	88 (33) 82 (23)	**19**	75 (44) 82 (23)	93 (26) 92 (14)	70 (46) 83 (20)
**A** **I**	**5**	47 (50) 60 (26)	81 (39) 76 (22)	68 (47) 52 (20)	**10**	73 (45) 70 (27)	83 (38) 75 (24)	56 (50) 56 (27)	**15**	66 (48) 50 (21)	89 (31) 74 (25)	80 (40) 59 (27)	**20**	69 (46) 69 (25)	77 (42) 86 (19)	50 (51) 76 (19)

		**Happiness**				**Gratitude**				**Guilt**				**Neutral**		
	**#**	**ENG *M* (*SD*)**	**POR *M* (*SD*)**	**GER *M* (*SD*)**	**#**	**ENG *M* (*SD*)**	**POR *M* (*SD*)**	**GER *M* (*SD*)**	**#**	**ENG *M* (*SD*)**	**POR *M* (*SD*)**	**GER *M* (*SD*)**	**#**	**ENG *M* (*SD*)**	**POR *M* (*SD*)**	**GER *M* (*SD*)**

**A** **I**	**21**	83 (38) 72 (24)	93 (45) 86 (19)	84 (37) 79 (22)	**26**	83 (38) 62 (25)	91 (28) 88 (17)	84 (37) 74 (20)	**31**	76 (43) 82 (22)	81 (40) 90 (16)	84 (37) 79 (22)	**36**	85 (36) 49 (32)	92 (27) 69 (37)	84 (37) 51 (36)
**A** **I**	**22**	81 (39) 78 (21)	90 (31) 90 (16)	98 (14) 79 (19)	**27**	83 (38) 68 (22)	92 (28) 87 (16)	84 (37) 70 (19)	**32**	75 (44) 78 (23)	72 (45) 93 (12)	86 (35) 84 (20)	**37**	80 (41) 56 (33)	72 (45) 66 (35)	78 (42) 40 (35)
**A** **I**	**23**	80 (41) 70 (26)	91 (28) 86 (17)	90 (30) 80 (20)	**28**	80 (41) 70 (22)	94 (24) 88 (17)	84 (37) 77 (17)	**33**	73 (45) 78 (32)	76 (43) 92 (15)	78 (42) 70 (27)	**38**	75 (44) 54 (35)	90 (31) 65 (36)	90 (30) 42 (33)
**A** **I**	**24**	90 (31) 70 (24)	79 (41) 86 (18)	76 (43) 76 (19)	**29**	76 (43) 68 (22)	90 (31) 81 (20)	84 (37) 76 (18)	**34**	68 (47) 89 (19)	77 (42) 89 (16)	82 (39) 87 (17)	**39**	80 (41) 44 (30)	65 (48) 65 (34)	88 (33) 41 (37)
**A** **I**	**25**	73 (45) 66 (24)	87 (34) 81 (21)	76 (43) 70 (25)	**30**	69 (46) 72 (23)	78 (42) 86 (16)	80 (40) 73 (18)	**35**	39 (49) 87 (15)	68 (47) 95 (11)	66 (48) 88 (18)	**40**	75 (44) 55 (33)	88 (33) 66 (37)	80 (40) 43 (34)

*EMO, emotion; ENG, English; POR, Portuguese; GER, German; M, mean; SD, standard deviation; A, recognition rate; I, intensity rate.*

#### Cluster Analyses

**FIGURE 1:**
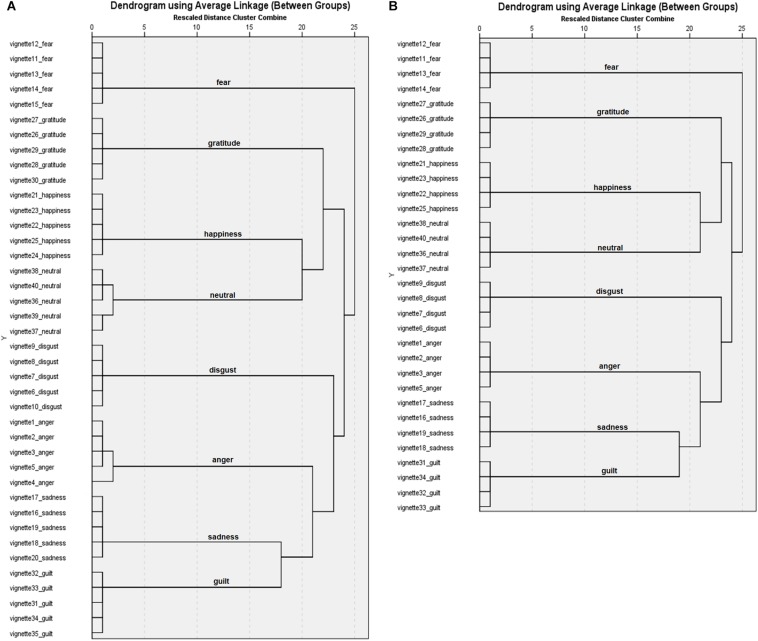
Dendrograms for the Portuguese vignettes with **(A)** 40 vignettes and **(B)** 32 vignettes.

Results (Figure 1A) from the hierarchical cluster analysis showed that for 6 emotion categories (disgust, fear, sadness, happiness, gratitude, and guilt) all 5 emotion vignettes for the target emotion categories were clustered together on the first cluster level. For 2 categories (neutral and anger), clusters emerged on first, and second level. After eliminating the vignette with the lowest recognition rates for each of the 8 emotion categories, cluster analysis including 32 vignettes showed 8 clusters including 4 vignettes each at the first level (Figure 1B). The single solution of 8 clusters also grouped all vignettes according to their target emotion. All following results are based on the 4 identified vignettes per emotion category.

#### Raw Hit Rates per Emotion Category

**FIGURE 2:**
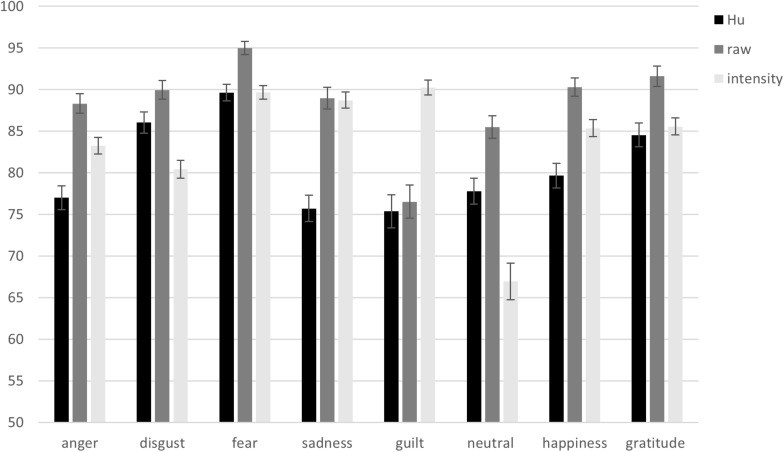
Unbiased hit rates (*Hu*), raw hit rates, and intensity rates from the Portuguese vignettes validation per emotion category. Error bars represent standard errors of the means.

Raw hit rates (*M*s and *SE*s) for the emotion categories (anger, disgust, fear, sadness, guilt, neutral, happiness, and gratitude) are presented in Figure 2.

#### Hu Rates per Emotion Category

*Hu* rates (*M*s and *SE*s) for the emotion categories (anger, disgust, fear, sadness, guilt, neutral, happiness, and gratitude) are presented in Figure 2. The confusions between emotion categories underlying the *Hu* rates are presented in Table 2.

**TABLE 2:** Confusions between the emotion categories in percentages from all three studies.

		Responses							
		Anger	Disgust	Fear	Sadness	Happiness	Neutral	Gratitude	Guilt
**Portuguese**									
Target emotion	Anger	88	3	1	6	0	2	0	0
	Disgust	2	90	1	2	1	4	0	0
	Fear	2	0	95	2	0	0	0	0
	Sadness	3	0	4	89	1	2	0	1
	Happiness	0	0	0	0	90	3	6	0
	Neutral	0	0	1	0	9	86	4	0
	Gratitude	0	1	0	0	5	2	92	0
	Guilt	9	2	1	11	0	1	0	77
**English**									
Target emotion	Anger	69	6	10	3	3	4	1	3
	Disgust	2	81	2	1	3	6	3	2
	Fear	2	0	86	1	2	3	2	3
	Sadness	2	3	5	78	3	3	3	4
	Happiness	1	2	2	0	83	3	7	1
	Neutral	1	1	1	1	9	80	5	3
	Gratitude	1	3	3	1	4	4	81	4
	Guilt	3	2	4	10	3	2	3	73
**German**									
Target emotion	Anger	88	0	5	1	0	4	0	1
	Disgust	1	78	1	3	4	11	0	0
	Fear	6	0	89	0	0	3	0	0
	Sadness	9	0	8	74	0	4	0	4
	Happiness	0	0	0	1	90	3	6	0
	Neutral	0	0	0	0	10	86	2	1
	Gratitude	0	0	0	1	11	3	84	0
	Guilt	4	0	1	6	1	4	0	83

*All values are based on four vignettes per target emotion category. The percentages in the diagonal line represent the raw hit rates; all percentages below and above the diagonal represent the percentages of confusions.*

#### Intensity Rates per Emotion Category

Intensity rates (*M*s and *SE*s) for the emotion categories (anger, disgust, fear, sadness, guilt, neutral, happiness, and gratitude) are presented in Figure 2.

### English Vignettes

Table 1 displays the *M*s and *SD*s of the raw hit rates and intensity rates for the individual vignettes.

#### Cluster Analyses

**FIGURE 3:**
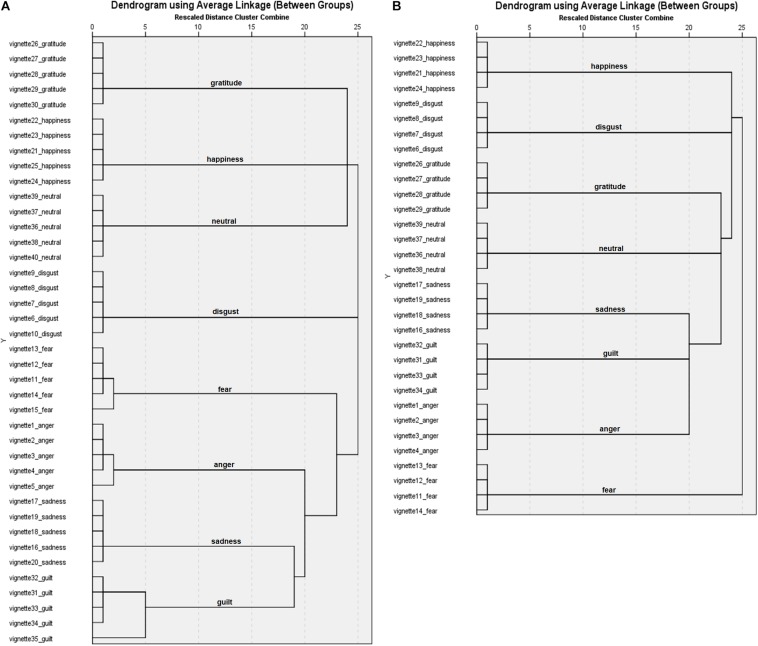
Dendrograms for the English vignettes with **(A)** 40 vignettes and **(B)** 32 vignettes.

Results (Figure 3A) from the hierarchical cluster analysis showed that for 5 emotion categories (disgust, sadness, gratitude, happiness, and neutral) all 5 emotion vignettes for the target emotion categories were clustered together on the first cluster level. For 3 categories (fear, anger, and guilt), 4 vignettes were categorised as belonging together on the first cluster level and 1 vignette was clustered to the target category on higher levels (level 2 and level 5). After eliminating the vignette with the lowest recognition rates for each of the 8 emotion categories, cluster analysis including 32 vignettes showed 8 clusters including 4 vignettes each at the first cluster level (Figure 3B). The single solution of 8 clusters also grouped all vignettes according to their target emotion. All following results are based on the 4 identified vignettes per emotion category.

#### Raw Hit Rates per Emotion Category

**FIGURE 4:**
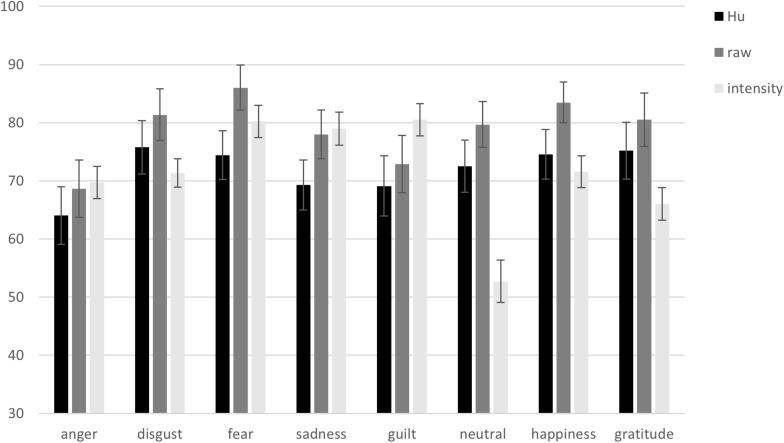
Unbiased hit rates (*Hu*), raw hit rates, and intensity rates from the English vignettes validation per emotion category. Error bars represent standard errors of the means.

Raw hit rates (*M*s and *SE*s) for the emotion categories (anger, disgust, fear, sadness, guilt, neutral, happiness, and gratitude) are presented in Figure 4.

#### Hu Rates per Emotion Category

*Hu* rates (*M*s and *SE*s) for the emotion categories (anger, disgust, fear, sadness, guilt, neutral, happiness, and gratitude) are presented in Figure 4. The confusions between emotion categories underlying the *Hu* rates are presented in Table 2.

#### Intensity Rates per Emotion Category

Intensity rates (*M*s and *SE*s) for the emotion categories (anger, disgust, fear, sadness, guilt, neutral, happiness, and gratitude) are presented in Figure 4.

### German Vignettes

Table 1 displays the *M*s and *SD*s of the raw hit rates and intensity rates for the individual vignettes.

#### Cluster Analyses

**FIGURE 5:**
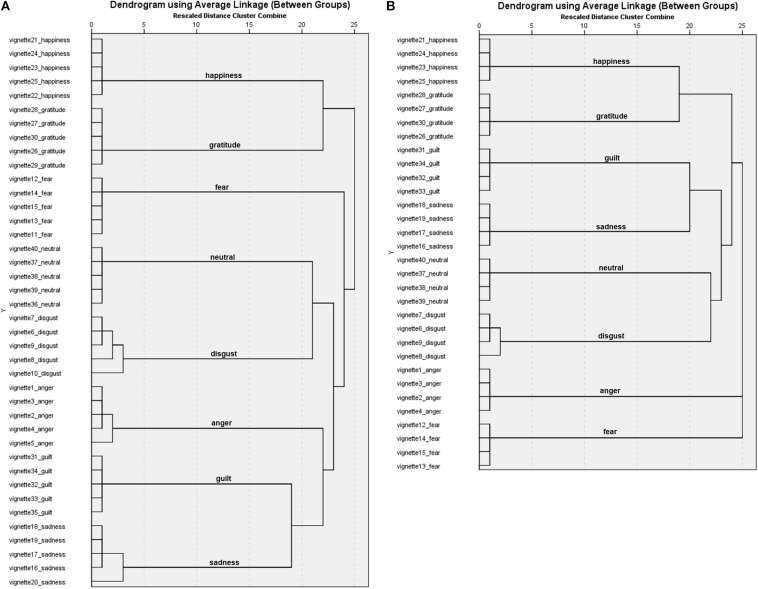
Dendrograms for the German vignettes with **(A)** 40 vignettes and **(B)** 32 vignettes.

Results (Figure 5A) from the cluster analysis showed that for 5 emotion categories (fear, gratitude, happiness, guilt, and neutral) all 5 emotion vignettes for the target emotion categories were clustered together. For 2 categories (sadness and anger), 4 stories were categorised as belonging together on the first cluster level and one story was clustered to the target emotion at a higher level (level 2 and level 3). For the category of disgust, 3 clusters emerged ranging from level 1 to 3. After eliminating the vignette with the lowest recognition rates for each of the 8 emotion categories, cluster analysis including 32 vignettes showed 7 clusters including 4 vignettes each at the first level and there was a second cluster between disgust vignettes at the second level (Figure 5B). The single solution of 8 clusters grouped all vignettes according to their target emotion including disgust. All following results are based on the 4 identified vignettes per emotion category.

#### Raw Hit Rates per Emotion Category

**FIGURE 6:**
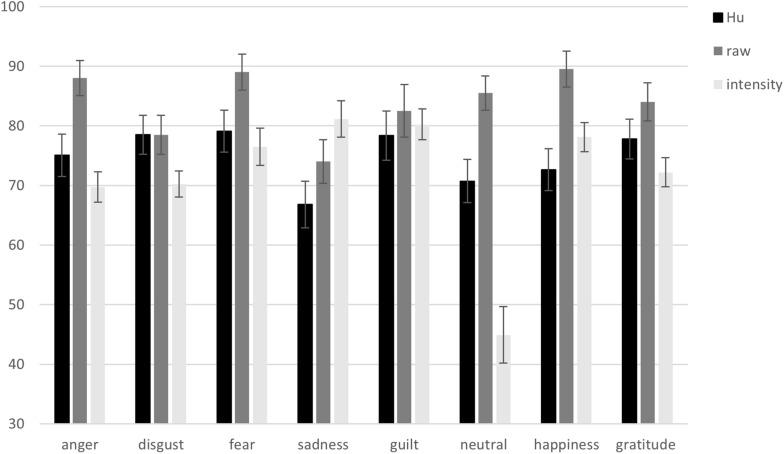
Unbiased hit rates (*Hu*), raw hit rates, and intensity rates from the German vignettes validation per emotion category. Error bars represent standard errors of the means.

Raw hit rates (*M*s and *SE*s) for the emotion categories (anger, disgust, fear, sadness, guilt, neutral, happiness, and gratitude) are presented in Figure 6.

#### Hu Rates per Emotion Category

*Hu* rates (*M*s and *SE*s) for the emotion categories (anger, disgust, fear, sadness, guilt, neutral, happiness, and gratitude) are presented in Figure 6. The confusions between emotion categories underlying the *Hu* rates are presented in Table 2.

#### Intensity Rates per Emotion Category

Intensity rates (*M*s and *SE*s) for the emotion categories (anger, disgust, fear, sadness, guilt, neutral, happiness, and gratitude) are presented in Figure 6.

## Discussion

The current research aimed at developing and validating verbal vignettes portraying short scenarios related to the specific emotions of anger, disgust, sadness, fear, happiness, gratitude, guilt, and neutral. Results showed that the individual emotion vignettes included in the final stimulus sets clearly mapped onto distinct emotion categories for each of the three languages. Results further showed high intensity rates for the self-reported experience of emotions while participants immersed themselves in the scenarios depicted in the vignettes. The vignettes can thus be considered successfully validated making them applicable within emotion research, e.g., emotion recognition and emotion elicitation.

When including five vignettes per emotion category, the results from the cluster analyses slightly exceeded the expected 8-cluster-solution. However, requesting a single solution with 8 clusters grouped all vignettes according to their target emotion. To only include the most similar vignettes per emotion category, the vignette with the lowest hit rate per emotion category was excluded which led to one cluster per included emotion category for the Portuguese and English stimulus set in subsequent analyses. The German stimulus set included one second level cluster, because one disgust vignette did not reach as high disgust attributions as the other three disgust vignettes. However, the additional cluster occurred at the second level and between disgust vignettes themselves; the next cluster only occurred at the 22nd level. The single solution with specified 8 clusters again grouped all vignettes according to their target emotion. It can be concluded that the final stimulus set of 32 emotion vignettes includes the most distinct stimuli which map clearly onto specific emotion categories for all three languages. As it is general practice to include example stimuli in psychological research with the aim to familiarise participants with the task procedures, the 8 excluded emotion vignettes with the lowest hit rates per emotion category could be used for such purposes.

The individual dendrograms further showed that some emotion categories were more similar to each other than others. For example, the emotion categories of happiness and gratitude were positioned closer to each other than categories such as anger, guilt, and sadness, while anger was positioned a little farther from the other emotion categories. It seems as though emotion categories positive in valence and emotion categories negative in valence were each positioned closer together. In addition, emotions with higher arousal level were positioned closer to each other than such of low arousal. Such a structure is in line with emotion theories such as the circumplex model of affect (Russell, 1980) defining emotions as representable on valence and arousal dimensions. When representing emotions in the two-dimensional space on valence and arousal, then negative emotion categories low in arousal are closer to each other (e.g., guilt and sadness) than to positive valence emotions that are low in arousal (e.g., happiness and gratitude), which themselves are closer to each other. It is interesting to note that the clustering in the current research was based on emotion label attributions of the emotion experienced while participants read scenarios rather than evaluations of the vignettes, e.g., on similarity. These results suggest that even when semantic understanding is necessary and a more cognitive approach to emotion elicitation is taken, the structure of emotion is represented. That is, it is more likely for participants to experience an emotion that is neighbouring the target emotion if it was not the target emotion that was experienced.

There were a few differences next to overlap between the three languages in terms of which individual emotion vignette per emotion category achieved the lowest hit rates (and was excluded from the main stimulus set per language). The neutral vignette with the lowest hit rate was different for all three languages. The lowest hit rate for anger and happiness vignettes were the same for the German and the English sample but not the Portuguese sample. However, the same vignettes led to lowest hit rates in all three languages for the emotion categories of fear, disgust, sadness, gratitude, and guilt. With many emotion categories overlapping in terms of which vignette had the lowest hit rate, this shows some consistency between the stimulus sets of the three languages.

The raw hit rates per emotion category were generally high and ranged between ∼75 and 95% in the Portuguese-speaking sample, ∼70–85% in the English-speaking sample, and ∼75–90% in the German-speaking sample. Even after correcting for response biases, the unbiased hit rates remained high in all three languages lowering the raw hit rates by roughly 5–10% per emotion category. Since there are no published verbal vignette stimulus sets including a similar number of emotion categories and the number of answer choices affects hit rates, the hit rates from the present stimulus sets cannot be directly compared to other stimulus sets. Nonetheless, these high agreement rates suggest that the stimulus sets in all three languages would be suitable for application in emotion recognition research. High agreement on participants’ reports about the emotion they experienced while immersing themselves into the scenarios described in the vignettes are also a prerequisite for applicability of the vignettes as valid emotion elicitation instrument.

The self-reported felt intensity reached medium to high intensities per emotion category suggesting that the vignettes are suitable for emotion elicitation. There were slight differences between the three languages regarding the intensity rates. The intensity rates (including the neutral category) in the Portuguese-speaking sample were ∼65–90%, ∼50–80% in the English-speaking sample, and ∼45–80% in the German-speaking sample. These results are only comparable to published film-based stimulus sets applicable for eliciting specific emotions, since no verbal vignette stimulus set is published presenting intensity ratings. Gross and Levenson (1995) reported between 37 and 64% intensity of felt emotions for the emotion categories included in their video stimulus set. The results from the vignettes presented here compare favourably to this stimulus set. The here obtained ranges of emotion intensity are below ceiling and thus allow for experimental manipulations aiming at investigating subsequent effects on emotion experience. For example, a study conducted in our laboratory showed that affiliative touch can modulate the evaluation of affective images (Wingenbach et al., unpublished). The created stimulus set could be used to investigate the effect of touch on emotion experience. Together, the created vignettes constitute a promising stimulus set for emotion elicitation.

There were differences in the hit rates between the three samples and the Portuguese sample achieved the highest hit rates across emotion categories. The samples differed from each other in their demographic characteristics, which can likely explain the differences in hit rates. The Portuguese sample included only university students who are required to participate in research as part of their degree and thus might have had prior experience with tasks as the current one. Better task performance by university students is often observed compared to general population samples and might also apply to the current research. In addition, the student sample included younger participants than the general population samples and the vignettes were written by age-similar peers. It is possible that these factors contributed to the higher hit rates in the Portuguese sample. Due to the differences between the samples, statistical comparisons of the results between the samples were not conducted.

In conclusion, three stimulus sets containing 32 vignettes (4 vignettes for each category of anger, disgust, fear, sadness, happiness, gratitude, guilt, and neutral) and an additional practice vignette per category were created and validated in three languages (Portuguese, English, and German) and the results suggest their suitability for emotion recognition and emotion elicitation research. The vignettes can be used for research purposes and are available to researchers free of charge downloadable from the Supplementary Material.

## Ethics Statement

This study was carried out in accordance with the recommendations of the Mackenzie Presbyterian University Ethics Committee’ with written informed consent from all subjects. All subjects gave written informed consent in accordance with the Declaration of Helsinki. The protocol was approved by the Mackenzie Presbyterian University Ethics Committee’.

## Author Contributions

PB conceptualised the study. LM and AH wrote the vignettes and collected the data. TW performed the data analysis and wrote the first version of the manuscript. All authors contributed to the data interpretation, manuscript writing, and approved the final version of the manuscript for submission.

## Conflict of Interest Statement

The authors declare that the research was conducted in the absence of any commercial or financial relationships that could be construed as a potential conflict of interest.

## Supplementary Material

TABLE S1 All 40 vignettes for each of the 3 languages.
